# Chronic ethanol increases systemic TLR3 agonist-induced neuroinflammation and neurodegeneration

**DOI:** 10.1186/1742-2094-9-130

**Published:** 2012-06-18

**Authors:** Liya Qin, Fulton T Crews

**Affiliations:** 1 Bowles Center for Alcohol Studies, School of Medicine CB 7178, UNC-CH, Chapel Hill, NC, 27599, USA

**Keywords:** Alcohol, toll-like receptor 3, oxidative stress, NADPH oxidase, neurodegeneration

## Abstract

**Background:**

Increasing evidence links systemic inflammation to neuroinflammation and neurodegeneration. We previously found that systemic endotoxin, a TLR4 agonist or TNFα, increased blood TNFα that entered the brain activating microglia and persistent neuroinflammation. Further, we found that models of ethanol binge drinking sensitized blood and brain proinflammatory responses. We hypothesized that blood cytokines contribute to the magnitude of neuroinflammation and that ethanol primes proinflammatory responses. Here, we investigate the effects of chronic ethanol on neuroinflammation and neurodegeneration triggered by toll-like receptor 3 (TLR3) agonist poly I:C.

**Methods:**

Polyinosine-polycytidylic acid (poly I:C) was used to induce inflammatory responses when sensitized with D-galactosamine (D-GalN). Male C57BL/6 mice were treated with water or ethanol (5 g/kg/day, i.g., 10 days) or poly I:C (250 μg/kg, i.p.) alone or sequentially 24 hours after ethanol exposure. Cytokines, chemokines, microglial morphology, NADPH oxidase (NOX), reactive oxygen species (ROS), high-mobility group box 1 (HMGB1), TLR3 and cell death markers were examined using real-time PCR, ELISA, immunohistochemistry and hydroethidine histochemistry.

**Results:**

Poly I:C increased blood and brain TNFα that peaked at three hours. Blood levels returned within one day, whereas brain levels remained elevated for at least three days. Escalating blood and brain proinflammatory responses were found with ethanol, poly I:C, and ethanol-poly I:C treatment. Ethanol pretreatment potentiated poly I:C-induced brain TNFα (345%), IL-1β (331%), IL-6 (255%), and MCP-1(190%). Increased levels of brain cytokines coincided with increased microglial activation, NOX gp91^phox^, superoxide and markers of neurodegeneration (activated caspase-3 and Fluoro-Jade B). Ethanol potentiation of poly I:C was associated with ethanol-increased expression of TLR3 and endogenous agonist HMGB1 in the brain. Minocycline and naltrexone blocked microglial activation and neurodegeneration.

**Conclusions:**

Chronic ethanol potentiates poly I:C blood and brain proinflammatory responses. Poly I:C neuroinflammation persists after systemic responses subside. Increases in blood TNFα, IL-1β, IL-6, and MCP-1 parallel brain responses consistent with blood cytokines contributing to the magnitude of neuroinflammation. Ethanol potentiation of TLR3 agonist responses is consistent with priming microglia-monocytes and increased NOX, ROS, HMGB1-TLR3 and markers of neurodegeneration. These studies indicate that TLR3 agonists increase blood cytokines that contribute to neurodegeneration and that ethanol binge drinking potentiates these responses.

## Background

Neuroinflammation linked to neuro- and psychopathology primarily involves induction of innate immune genes expressed in microglia, the brain monocyte-like innate immune cells. We previously found that endotoxin induced or injection of TNFα increased blood and brain TNF α activated microglia and induced brain monocyte chemotactic protein-1(MCP-1) (also known as chemokine (C-C motif) ligand 2 (CCL2)), IL-1β and TNFα mRNA that led to persistent neuroinflammation and a delayed (seven to ten months) loss of substantia nigra tyrosine hydroxylase-positive dopamine neurons [1]. These findings were extended to studies of motor function, which found that seven months after a single endotoxin dose, L-3,4-dihydroxyphenylalanine (L-DOPA) reversible rotorod deficits appeared, with continuing loss of function with age and loss of substantia nigra neurons [2]. Endotoxin (lipopolysaccharide (LPS)) activates innate immune responses through toll-like receptor 4 (TLR4) activation of nuclear factor-kappa B (NF-κB) transcription of proinflammatory gene transcription within microglia and other cells. Microglia and proinflammatory signals include multiple positive loops of autocrine and paracrine amplification that contribute to persistent microglial activation in brain [3]. These findings are consistent with hypotheses that infections early in life impact overall life span [4,5] and that microglial activation contributes to age-associated neurodegenerative diseases [6]. These hypotheses suggest that systemic inflammatory responses contribute to chronic diseases. Although multiple toll-like receptors activate monocyte-microglial proinflammatory responses, most studies have modeled bacterial endotoxin-LPS-TLR4-induced brain responses. We hypothesized that toll-like receptor 3 (TLR3), a receptor that activates monocyte NF-κB transcription of proinflammatory cytokines in response to virus-like mRNA [7], would induce blood proinflammatory responses and brain neuroinflammation.

Polyinosine-polycytidylic acid (poly I:C) is a synthetic double-stranded RNA that with endogenous co-agonists, such as high-mobility group box (HMGB) proteins, stimulates proinflammatory innate immune responses through TLR3 [8]. TLR3 receptors activate NF-κB in monocyte-microglia, astrocytes and other cells [7,9-14] and increase proinflammatory cytokine expression and neuroinflammation [15-18]. Although low levels of TLR3 are expressed in healthy human brains, multiple neurodegenerative diseases show increased expression of TLR3 receptors across brain regions [19]. Recent studies indicate that TLR receptors and endogenous agonist respond to cell stress, excessive glutamate excitation and/or other ‘danger’ signals [20,21]. For example, high-mobility group box 1 (HMGB1), an agonist at multiple TLR receptors and required for TLR3 activation [22], is released from cells by neurotransmitters including glutamate, proinflammatory cytokines and many other stimuli that amplify proinflammatory responses [3]. TLR3 may also play a role in neuroplasticity since TLR3-deficient mice have increased hippocampal neurogenesis and altered cognition [23]. These findings suggest that levels of brain HMGB1 and TLR receptors contribute to brain function and neuroinflammation.

Alcohol (ethanol) is a common dietary constituent that impacts heath. Heavy binge drinking increases mortality by escalating the risk of multisystem diseases in peripheral organs as well as psychiatric and neurological disorders in the central nervous system (CNS) [24]. Heavy alcohol drinkers have elevated levels of C-reactive protein, an innate immune marker [18,23]. Previously, we reported that levels of MCP-1, markers of microglia and NOX gp91^phox^ were significantly increased in human postmortem alcoholic brain, compared to human moderate drinking control brain [25,26]. Further, we found that human postmortem alcoholic brain has increased histochemical markers of neuronal cell death [26]. Alcoholism is known to cause neurodegeneration [9]. We found that chronic administration of ethanol to mice increased brain and liver cytokines and chemokines, including TNFα, IL-1β and MCP-1 [27]. Although acute ethanol has been found to inhibit proinflammatory TLR responses, including the TLR3 agonist poly I:C [28,29], recent studies have found that toll-like receptors (TLRs) contribute to ethanol activation of brain proinflammatory responses and neurodegeneration [30]. These studies support a link among neuroinflammation, ethanol and systemic proinflammatory responses.

Here, we report that acute TLR3 agonist, poly I:C, systemic administration increases blood and brain TNFα, IL-1 β, IL-6, and MCP-1. Brain responses persist for at least three days, whereas blood levels return to controls by one day. Chronic ethanol treatment causes mild increases in blood and brain, whereas sequential ethanol-poly I:C treatment leads to large responses, with increases in blood and brain proinflammatory responses across treatment groups. Increased levels of brain cytokines coincided with activated microglial morphology, increased NOX gp91^phox^, superoxide and markers of neuronal cell death, for example, activated caspase-3 and Fluoro-Jade B staining. Ethanol potentiation of poly I:C was associated with ethanol-induced TLR3 and HMGB1 expression. Blocked microglial activation by minocycline and naltrexone blunted cell death markers. These studies suggest that the magnitude of systemic proinflammatory responses contribute to the magnitude of microglial activation, brain neuroinflammation and neurodegeneration.

## Materials and methods

### Animals

Eight-week-old male (20 to 22 g) C57BL/6 mice were purchased from Jackson Laboratories (Bar Harbor, ME, USA). All protocols and procedures in this study were approved by the Institutional Animal Care and Use Committee (IACUC) and were in accordance with the National Institute of Health regulations for the care and use of animals in research.

### Reagents

Poly I:C was purchased from Amersham Biosciences (Piscataway, NJ, USA). Goat polyclonal TLR3 (N-14) and HMGB1 (K-12) antibodies were purchased from Santa Cruz Biotechnology, Inc. (Santa Cruz, CA, USA). Rabbit anti-Iba1 antibody was purchased from Wako Pure Chemical Industries, Ltd. (Osaka, Japan). Monoclonal anti-mouse gp91^phox^ was from Transduction Laboratories (Lexington, KY, USA). Rabbit polyclonal MAP-2 antibody was purchased from Abcam (Cambridge, MA, USA). Polyclonal rabbit anti-glial fibrillary acidic protein antibody was from DakoCytomation (Glostrup, Denmark). Cleaved caspase-3 (Asp175) antibody was from Cell Signaling Technology (Danvers, MA, USA). Fluoro-Jade B and mouse NeuN antibody were from Chemicon International (Temecula, CA, USA). TNFα, IL-1β, MCP-1 and IL-6 ELISA kits were purchased from R&D Systems Inc. (Minneapolis, MN, USA). Hydroethidine was from Invitrogen Molecular Probes (Eugene, OR, USA). All other reagents came from Sigma-Aldrich Chemical Company (St. Louis, MO, USA).

### Drug treatments

**Time course of TNFα response to TLR3 agonist poly I:C:** Male C57BL/6 mice were intraperitoneally (i.p.) injected with a single dose of poly I:C (250 μg/kg) and d-(+) galactosamine hydrochloride (d-GalN 20 mg/kg), or saline (control). Mice were sacrificed at selected time points, and sera and brain samples were used for TNFα measurement by ELISA.

**Effect of prior ethanol exposure on poly I:C-induced neuroinflammation and neurodegeneration:** Male C57BL/6 mice were treated with water or ethanol (5 g/kg, intragastrically (i.g.), 25% ethanol w/v) daily for 10 days. The average blood alcohol concentration at one hour after the first ethanol treatment and the last ethanol treatment was 291 mg/dl ± 16 (w/v, n = 10) and 301 mg/dl ± 19 (w/v, n = 10), respectively. Twenty-four hours after the last ethanol administration, mice were injected with either saline (control) or poly I:C (250 μg/kg, i.p.) and d-GalN (20 mg/kg, i.p.) in saline. Mice were sacrificed three hours after poly I:C treatment. Gene expression and protein synthesis of proinflammatory cytokines, oxidative enzymes, microglial morphology, and neurodegeneration were examined using real-time PCR, ELISA, and immunohistochemistry.

**Effect of minocycline and naltrexone on ethanol-poly I:C-induced microglial activation and caspase-3 expression:** Male C57BL/6 mice were treated with water or ethanol (5 g/kg, i.g.) daily for 10 days. Mice were then injected intraperitoneally with saline, poly I:C (250 μg/kg) and d-GalN (20 mg/kg) in saline 24 hours after the last dose of ethanol. For ethanol-poly I:C-minocycline or naltrexone group, mice were injected with minocycline (50 mg/kg, i.p.) or naltrexone (60 mg/kg, i.p.) 30 minutes before every dose of ethanol or poly I:C treatment. Brain samples were collected three hours after poly I:C and d-GalN administration. All experiments were performed with seven mice per group and repeated two times.

### Real-time PCR analysis

Total RNA was extracted from the brain samples of mice treated with ethanol, poly I:C, ethanol-poly I:C or saline, and reverse transcribed as described previously [31]. The primer sequences used in this study were as follows: TNFα, 5′-GAC CCT CAC ACT CAG ATC ATC TTC T-3′ (forward) and 5′-CCT CCA CTT GGT GGT TTG CT-3′ (reverse); IL-1β, 5′-CTG GTG TGT GAC GTT CCC ATT A-3′ (forward) and 5′-CCG ACA GCA CGA GGC TTT-3′ (reverse); IL-6, 5′-GGC CTT CCC TAC TTC ACA AG-3′ (forward) and 5′-ATT TCC ACG ATT TCC CAG AG-3′ (reverse); MCP-1, 5′-ACT GAA GCC AGC TCT CTC TTC CTC-3′ (forward) and 5′-ACT GAA GCC AGC TCT CTC TTC CTC-3′ (reverse); TLR3, 5′-TTG TCT TCT GCA CGA ACC TG-3′ (forward) and 5′-GGC AAC GCA AGG ATT TTA TT-3′ (reverse); HMGB1, 5′-CCA TTG GTG ATG TTG CAA AG-3′ (forward) and 5′-CTT TTT CGC TGC ATC AGG TT-3′ (reverse); gp91^phox^, 5′-CAG GAG TTC CAA GAT GCC TG-3′ (forward) and 5′-GAT TGG CCT GAG ATT CAT CC-3′ (reverse); β-actin, 5′-GTA TGA CTC CAC TCA CGG CAA A-3′ (forward) and 5′-GGT CTC GCT CCT GGA AGA TG-3′ (reverse). The SYBR green PCR master mix (Applied Biosystems, Foster City, CA, USA) was used for real-time PCR analysis. The relative differences in expression between groups were expressed using cycle time (Ct) values normalized with β-actin, and relative differences between control and treatment group were calculated and expressed as relative increases setting control as 100%.

### Immunohistochemistry

Mouse brains were fixed with 4% paraformaldehyde in phosphate buffered saline (PBS) and processed for immunostaining as described previously [31]. TLR3 and HMGB1 were immunostained with TLR3 and HMGB1 antibodies. Microglia were stained with rabbit anti-Iba1 antibody. NADPH oxidase membrane subunit gp91^phox^ was immunostained with monoclonal anti-mouse gp91^phox^ IgG. Activated caspase-3 was immunostained with polyclonal anti-cleaved caspase-3 antibody. Caspase-3 co-labeling with NeuN was performed with caspase-3 and NeuN antibodies. Neurons were stained with MAP2 or NeuN antibody. Astrocytes were labeled with GFAP antibody. Immunolabeling was visualized by using nickel-enhanced 3,3′-diaminobenzidinne (DAB) or Alexa Fluor 488 or 555 dye.

### O_2_^-^ and O_2_^-^-derived oxidant measurement

*In situ* visualization of O_2_^-^ and O_2_^-^-derived oxidant production was assessed by hydroethidine histochemistry [32,33]. Mice were injected with dihydroethidium (10 mg/kg, i.p.) in 0.5% carboxymethyl cellulose 2.5 hours after poly I:C. injection. Brains were harvested 30 minutes later and frozen sections (15 μm) were examined for hydroethidine oxidation product, ethidium accumulation, by fluorescence microscopy (excitation 510 nm; emission 580 nm).

### Fluoro-Jade B staining with NeuN labeling

Mouse brain sections were immunostained with mouse NeuN antibody. Immunolabeling was visualized by using Alexa Fluor 555 dye. Sections were rinsed three times with PBS and one time with water before performing the Fluoro-Jade B procedure. Briefly, sections stained with NeuN were mounted on Superfrost Plus microscope slides and air dried overnight. The sections were rinsed in distilled water for two minutes to rehydrate and transferred to a solution of 0.06% potassium permanganate for ten minutes. The sections were then rinsed in distilled water for two minutes and placed in a 0.0004% Fluoro-Jade B solution made by adding 4 ml of a 0.01% stock solution of Fluoro-Jade B to 96 ml of 0.1% acetic acid. After 20 minutes in the Fluoro-Jade B staining solution, the stained slides were thoroughly washed in distilled water, dehydrated, and cover slipped.

### Microscopic quantification

Immunoreactivity of mouse gp91^phox^ and Iba1, fluorescent intensity of Fluoro-Jade B and ethidium were quantified using Bioquant Image Analysis software (Nashville, TN, USA). Images were captured on an Olympus (Tokyo, Japan) BX51 microscope and Sony (Tokyo, Japan) DCX-390 video camera at 40X. Light levels were normalized to preset levels and the microscope, camera, and software were background corrected to ensure reliability of image acquisition [34]. In each region (cortex and dentate gyrus), six random images from each brain sample were captured within a standard region of interest (ROI), the density of immunostaining and fluorescence was measured in pixels within this area (pixels/mm^2^). Subsequently, the average of the six measurements was used to represent the immunoreactivity or fluorescence intensity of each sample. When measuring fluorescence intensity in the cells, we eliminated the background by adjusting the threshold to avoid background staining. For + immunoreactive (+IR) cell counting, a modified stereological method was used to quantify cells within regions of interest following immunostaining of brain sections using the CAST stereological system [35,36]. Specifically, cell density (N_v_) of TLR3, HMGB1, caspase-3 and Iba1 + immunoreactive (+IR) cells was determined following the optical dissector method [37,38], which was calculated as follows:

(1) $$N_{v}=\Sigma Q/\Sigma dis\sec tor\times A\left(fr\right)xh$$

Where ∑Q is the sum of + IR cells counted from each dissector frame, ∑dissector is the sum of the number of dissector frames counted, A(fr) is the known area associated with each dissector frame, and h is the known distance between two dissector planes (10 μm was used). For co-labeling study, double-stained sections were digitally photographed with Leica (Wetzlar, Germany) SP2-AOBS confocal microscope and analyzed with Leica SP2 LCS software.

### TNFα, IL-1β, IL-6, and MCP-1 assays

Frozen brains were homogenized in 100 mg tissue/ml cold lysis buffer (20 mM Tris, 0.25 M sucrose, 2 mM EDTA, 10 mM EGTA, 1% Triton X-100) and one tablet of Complete Mini protease inhibitor cocktail tablets/10 ml (Roche Diagnostics, Indianapolis, IN, USA). Homogenates were centrifuged at 100,000 × g for 40 minutes, supernatant was collected, and protein levels determined using the BCA protein assay reagent kit (PIERCE, Milwaukee, WI, USA). The levels of TNFα, IL-1β, IL-6 and MCP-1 in brains or sera were measured with TNFα, IL-1β, IL-6 and mouse JE/MCP-1 commercial enzyme-linked immunosorbent assay (ELISA) kits from R&D Systems (Minneapolis, MN, USA), as described previously [39].

### Statistical analysis

The data are expressed as mean ± standard error of the mean (SEM) and statistical significance was assessed with an ANOVA followed by Bonferroni’s *t* test using the StatView program (Abacus Concepts, Berkeley, CA, USA). A value of *P* <0.05 was considered statistically significant.

## Results

### TLR3 agonist induction of systemic and brain innate immune proinflammatory genes

**Figure 1:**
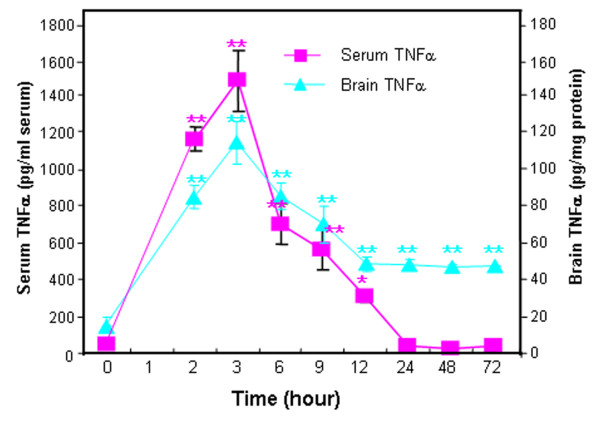
** TLR3 agonist poly I:C induction of TNFα in mouse serum and brain.** Levels of proinflammatory cytokine TNFα were determined following a single poly I:C (250 μg/kg, i.p.) and d-galactosamine (D-GalN, 20 mg/kg, i.p.) injection into C57BL/6 mice. At the time points indicated, mice were sacrificed and brain extracts and sera prepared as described in methods. Note both brain and serum TNFα peaked at three hours. Interestingly, blood (serum) TNFα declined to control level by 24 hours whereas brain TNFα level remained elevated at about half the peak level for at least 72 hours. The results shown are the means ± SEM of two experiments performed with seven mice per time point. *P <0.05, **P <0.01, compared to the corresponding vehicle controls.

We have previously found that induction of brain TNFα following intraperitoneal injections of LPS, a toll-like receptor 4 agonist, is related to blood TNFα that is transported into the brain inducing a response that lasted at least 10 months [1]. We hypothesized that poly I:C, a TLR3 agonist known to activate systemic and brain innate immune responses, would induce parallel systemic and brain proinflammatory responses that cause persistent brain activation. Poly I:C treatment of mice increased TNFα serum levels that peak around three hours at more than tenfold basal levels returning to near zero by 24 hours (Figure 1). Poly I:C treatment increased brain levels of TNFα that peaked at three hours after poly I:C at about 6 fold basal levels and remained significantly elevated for at least three days. These findings are consistent with acute systemic proinflammatory activation contributing to persistent brain neuroinflammatory responses.

**Figure 2:**
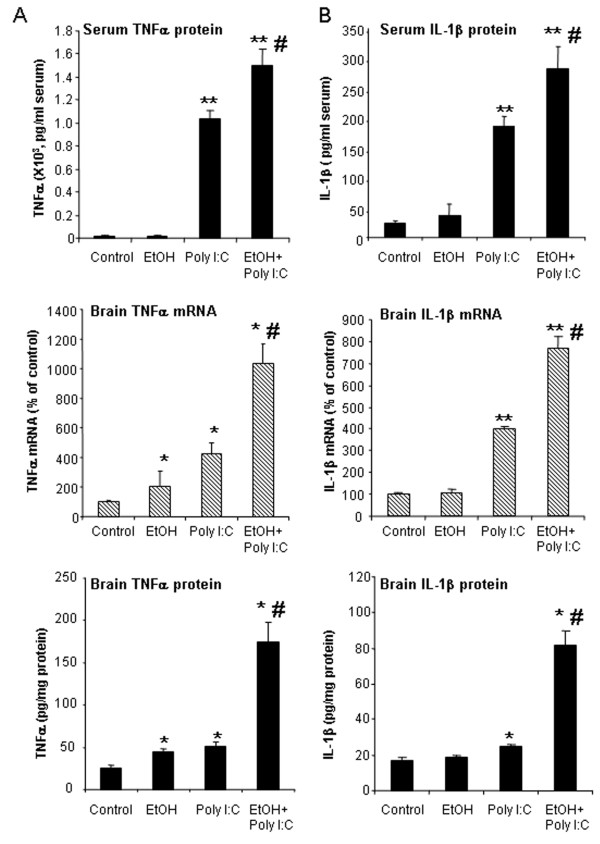
** Effect of chronic ethanol treatment on poly I:C-induced blood and brain TNFα and IL-1β.** As described in the methods, male C57BL/6 mice were treated intragastrically with ethanol (5 g/kg, i.g. daily for 10 days) and 24 hours after the last dose of ethanol treatment injected intraperitoneally with poly I:C (250 μg/kg) plus D-GalN (20 mg/kg). Brains were collected three hours after poly I:C injection for all groups, that is, ethanol alone is 27 hours after the last dose of ethanol. The levels of serum TNFα and IL-1β protein and brain TNFα and IL-1β mRNA and protein were measured by real-time PCR and ELISA. **(A)** Poly I:C treatment increased serum TNFα protein and brain TNFα mRNA and protein. Ethanol treatment did not alter serum TNFα protein, but increased brain TNFα mRNA and protein. Ethanol exposure potentiated poly I:C-induced serum TNFα protein as well as brain TNFα mRNA and protein. **(B)** Poly I:C treatment increased serum IL-1β protein and brain IL-1β mRNA and protein. Ethanol alone had no significant effect. Ethanol pretreatment potentiated poly I:C-induced serum IL-1β protein and brain IL-1β gene expression and protein synthesis. The results are the means ± SEM in two independent experiments with seven animals per group. *P <0.05, **P <0.01, compared with the vehicle control group. ^#^*P* <0.05, compared with the corresponding poly I:C treated group.

**Figure 3:**
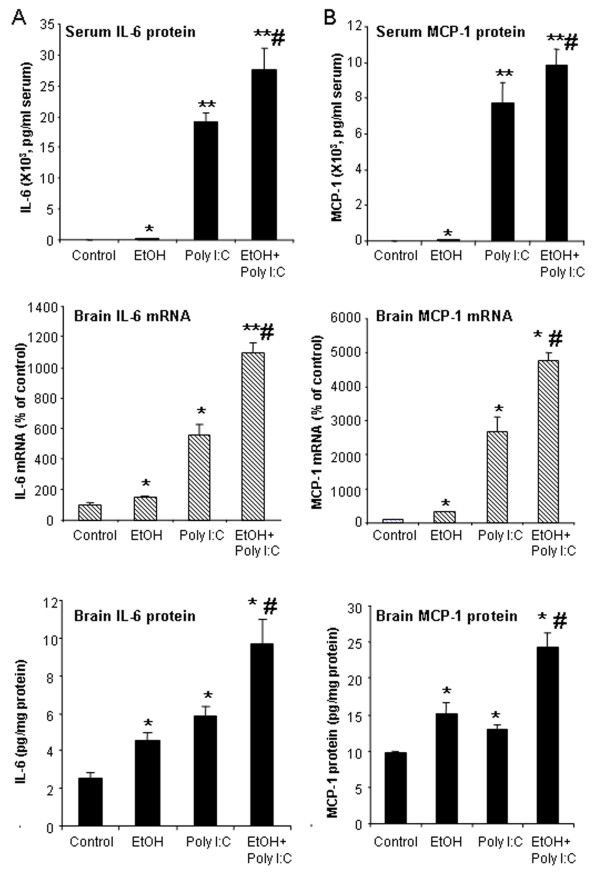
** Effect of chronic ethanol treatment on poly I:C-induced blood and brain IL-6 and MCP-1.** As described in the methods, male C57BL/6 mice were treated intragastrically with ethanol (5 g/kg, i.g. daily for 10 days) and 24 hours after the last dose of ethanol treatment injected intraperitoneally with poly I:C (250 μg/kg) plus D-GalN (20 mg/kg). Brains were collected three hours after poly I:C injection for all groups, that is, ethanol alone is 27 hours after the last dose of ethanol. **(A)** Ethanol or poly I:C alone treatment increased serum IL-6 protein and brain IL-6 mRNA and protein. Sequential ethanol-poly I:C treatment significantly augmented the blood and brain levels of IL-6. **(B)** Ethanol or poly I:C alone treatment increased serum MCP-1 protein and brain MCP-1 mRNA and protein. Ethanol pretreatment potentiated poly I:C-induced serum MCP-1 protein and brain MCP-1 gene expression and protein synthesis. The results are the means ± SEM in two independent experiments with seven animals per group. *P <0.05, **P <0.01, compared with the vehicle control group. ^#^*P* <0.05, compared with the corresponding poly I:C treated group.

To investigate chronic ethanol proinflammatory responses and poly I:C TLR3 agonist responses across multiple proinflammatory agents, we determined proinflammatory responses in ethanol alone, poly I:C alone or sequential ethanol-poly I:C administration in C57BL/6 mice. We compared induction of cytokines, TNFα, IL-1β, IL-6 and the chemokine, MCP-1, that we previously found increased in postmortem human alcoholic brain [25]. Brains of mice treated for 10 days with a binge-drinking dose of ethanol followed by 27 hours of abstinence showed a significant increase in both TNFα mRNA and protein, although TNFα did not show an elevation in serum after chronic ethanol (Figure 2). Brains of ethanol-treated mice also showed increased IL-6 and MCP-1 mRNA and protein. Serum of ethanol-treated mice showed a 4 fold increase in MCP-1 and a 50% increase in IL-6, although values remained relatively low compared to those found with poly I:C treatment (Figure 3). Poly I:C treatment increased serum levels of TNFα, IL-1β, IL-6, and MCP-1 manyfold over vehicle control basal levels. Similarly brain mRNA and protein for TNFα, IL-1β, IL-6, and MCP-1 were increased manyfold by poly I:C treatment (Figures 2 and 3). Interestingly, ethanol pretreatment potentiated poly I:C responses increasing levels of proinflammatory cytokines in both blood and brain. The blood IL-1β level increased more than 10 fold in ethanol-poly I:C-treated mice (Figure 2). In brain, ethanol pretreatment potentiated poly I:C induction of TNFα mRNA from 4 to 10 fold, IL-1β mRNA from 4 to 8 fold, IL-6 mRNA from 6 to 12 fold, and MCP-1 mRNA from 28 to 49 fold (Figures 2 and 3). Ethanol pretreatment also increased poly I:C induction of TNFα, IL-1β, IL-6, and MCP-1 protein levels in brain (Figures 2 and 3). These results indicate that acute serum proinflammatory responses mimic brain proinflammatory responses. Both blood and brain show a modest ethanol response, marked poly I:C TLR3 agonist response and sequential ethanol-poly I:C amplification of proinflammatory gene induction.

**Figure 4:**
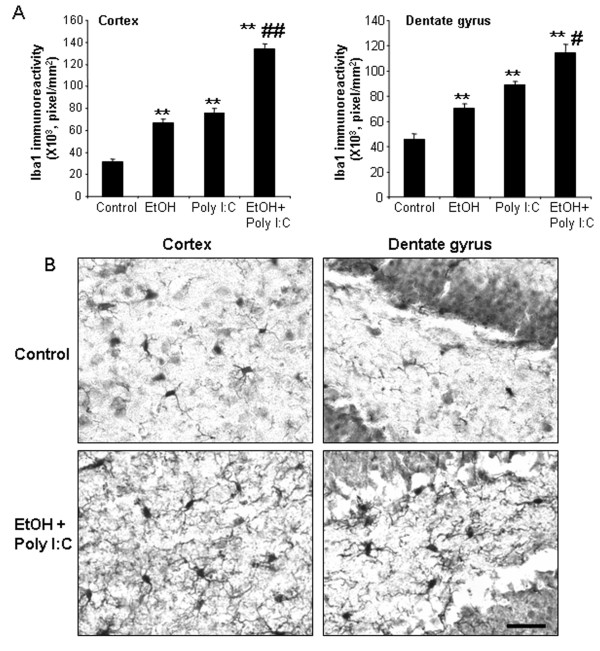
** Immunocytochemical analysis of microglia.** Mice were treated as described above. **(A)** Levels of immunoreactive density of Iba1, a marker of microglia, in cortex and hippocampal dentate gyrus were quantified using BioQuant image analysis software and presented as mean ± SEM in pixel/mm^2^. Ethanol alone, poly I:C alone and ethanol-poly I:C treated groups all show increased Iba1 + IR in both brain regions. **(B)** Representative images of Iba1 + IR cells in cortex and dentate gyrus from control and ethanol-poly I:C-treated groups. In water control group, microglia showed a resting morphological shape. In either ethanol or poly I:C alone-treated groups, some of the microglia are enlarged (images not shown). Iba1 + IR cells in EtOH-poly I:C-treated mouse brains have increased cell size, irregular shape, and intensified Iba1 staining consistent with morphological changes in activated microglia. Scale bar, 200 μm.

Microglia, the resident innate immune cells in the brain, produce proinflammatory factors that contribute to neurodegeneration through increased proinflammatory superoxide and other toxic agents [6]. In previous studies, we found that the postmortem human alcoholic brain showed increased Iba1 + IR, a microglial marker [25]. We investigated microglial Iba1 + IR to evaluate size and morphological changes of microglia. Control subjects showed resting microglial morphology (Figure 4), with mild increases in Iba1 + IR staining with ethanol or poly I:C alone treatment (images not shown). Ethanol pretreatment further potentiated poly I:C increased Iba1 + IR. Multiple brain regions, including cortex and dentate gyrus, showed increased Iba1 + IR (Figure 4). These studies indicate that ethanol primes microglia and potentiates TLR3 agonist poly I:C activation of microglia.

### Proinflammatory brain NADPH oxidase and reactive oxygen species

**Figure 5:**
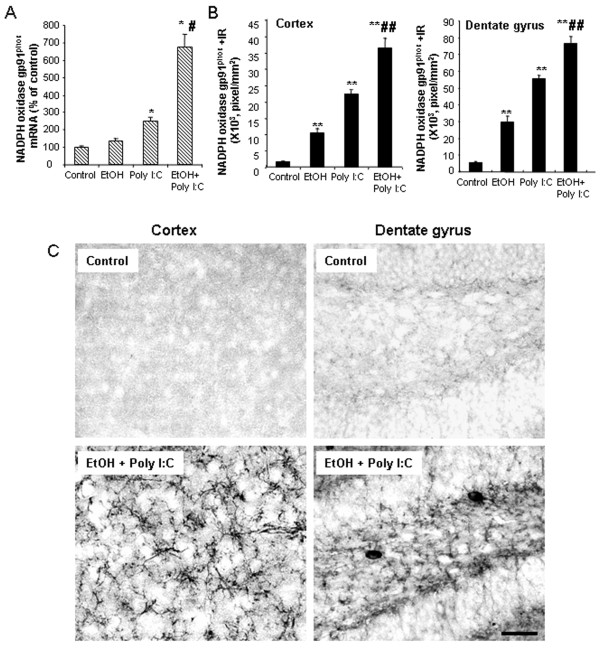
** Induction of NOX-NADPH oxidase subunit gp91**^**phox**^**expression.** Male C57BL/6 mice were treated with ethanol, poly I:C, ethanol-poly I:C as indicated in methods. **(A)** gp91^phox^ gene expression was determined by real-time PCR three hours after poly I:C treatment. Note chronic ethanol pretreatment increased brain poly I:C-induced gp91^phox^ mRNA by 2.7-fold. **(B)** NADPH oxidase subunit gp91^phox^ + IR in cortex and dentate gyrus (DG). Sections were stained with monoclonal mouse gp91^phox^ antibody and quantified by BioQuant image analysis system. NADPH oxidase subunit gp91^phox^ + IR was increased in cortex about 6 fold by ethanol and 14 fold by poly I:C and in DG about 5 fold by ethanol and 10 fold by poly I:C. Pretreatment of ethanol significantly enhanced poly I:C-induced gp91^phox^ + IR in both cortex and DG. **(C)** The images shown are representative of gp91^phox^ + IR cells from cortex (left) and dentate gyrus (right) for control (upper images) and ethanol-poly I:C groups (lower images). *P <0.05, **P <0.01, compared with the vehicle control mice. ^#^*P* <0.05, ^##^*P* <0.01, compared with poly I:C-treated mice. Scale bar, 200 μm.

**Figure 6:**
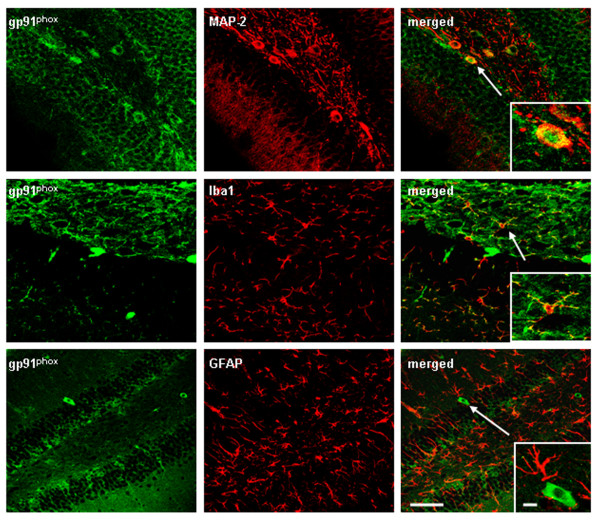
** Confocal microscopy with cell specific markers finds neuronal and microglial expression of NADPH oxidase subunit gp91**^**phox**^**.** Brain sections from ethanol-poly I:C-treated mice were double-labeled for gp91^phox^ in green with neuronal marker MAP-2, microglial marker Iba1, or astroglial marker GFAP in red. Co-labeling was investigated using a Leica SP2 LCS confocal microscope with associated software. The representative images shown are from dentate gyrus of mice treated with ethanol-poly I:C. The left panel of pictures shows gp91^phox^ + IR. The middle panel shows cell specific markers, for example, neuronal MAP-2 (upper panel), microglial Iba1 (middle) and astrocyte GFAP (lower panel) pictures. Merged images are to the right. Merged yellow indicates red and green are combined and likely co-localized within the marked cell. Merged pictures on the right with enlarged cells suggest that gp91^phox^ + IR is expressed in MAP-2 neurons (yellow) and Iba1 microglia (yellow), but not in astrocytes. Scale bar, 30 μm; inset 5 μm.

NADPH oxidase (NOX) is a family of oxidases known to produce superoxide and NOX is thought to be involved in neurodegeneration [40]. To determine the role of NOX in TLR3 agonist proinflammatory responses, we investigated the expression of NOX gp91^phox^, the catalytic subunit of phagocytic oxidase commonly associated with proinflammatory responses. Poly I:C induced NOX gp91^phox^ mRNA 2 to 3 fold (Figure 5A) and NOX gp91^phox^ + IR by manyfold more in cortex and hippocampal dentate gyrus (Figure 5B). Ethanol treatment produced a non-significant trend toward an increase in NOX gp91 mRNA, but did increase gp91^phox^ + IR above control levels to about half of that found with poly I:C alone. Ethanol potentiated the poly I:C TLR3 responses with both NOX gp91^phox^ mRNA and NOX gp91^phox^ + IR increased in cortex and hippocampus (Figure 5). Double antibody studies with cell specific markers and confocal microscopy indicate that ethanol-poly I:C-induced NOX gp91^phox^ + IR is colocalized with neuronal marker (MAP-2) and a microglial marker (Iba1) but there is little colocalization with astrocytic marker (GFAP) (Figure 6). These results indicate that TLR3 activation increases brain NOX gp91^phox^.

**Figure 7:**
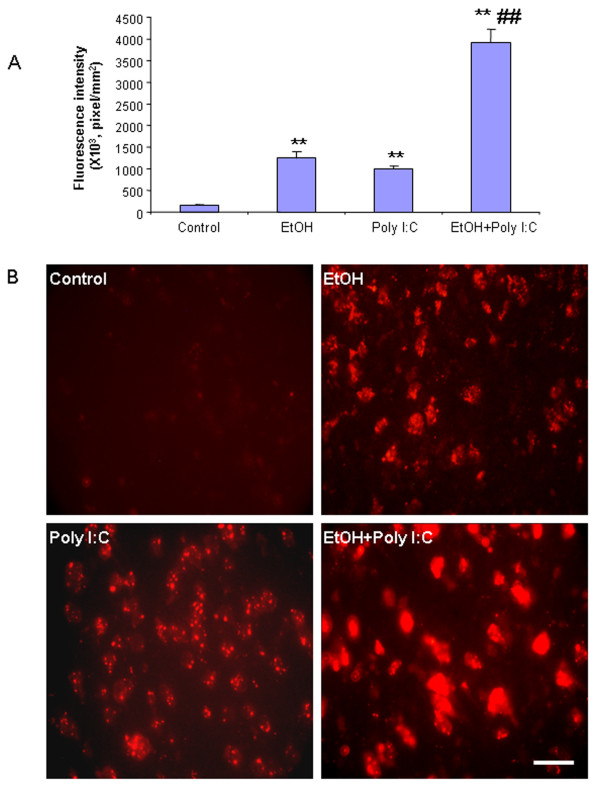
** Superoxide formation and oxidative stress in brain.** Mice were injected with hydroethidine (dihydroethidium, 10 mg/kg, i.p.) 2.5 hours after poly I:C treatment and brains harvested 30 minutes later, frozen and sectioned (15 μm thickness) as described in the methods. The oxidation product, ethidium, is formed from dihydroethidium by superoxide resulting in ethidium accumulation within cells producing superoxide. Ethidium is detected as red nuclei by fluorescence microscopy. The level of fluorescence intensity of ethidium-positive cells was quantified by BioQuant image analysis software. **(A)** Quantitation of ethidium fluorescence indicates ethanol, poly I:C and ethanol + poly I:C treatment significantly increases O_2_^-^ and O_2_^-^-derived oxidant production in cortex. **(B)** Representative images of ethidium fluorescence. Ethanol and poly I:C alone increased O_2_^-^ and O_2_^-^-derived oxidant production compared with vehicle control. Ethanol pretreatment significantly potentiated poly I:C-induced O_2_^-^ and O_2_^-^-derived oxidant production. **P <0.01, compared with vehicle control group. ^##^*P* <0.01 compared with poly I:C group. Scale bar, 200 μm.

Activation of NADPH oxidase (NOX) produces superoxide in brain and superoxide formation was assessed in the present experiment by histochemistry using *in situ* visualization of reactive oxygen species, for example, O_2_^-^ and O_2_^-^-derived oxidant production of ethidium from hydroethidine [32,33]. In vehicle-treated mice, there was little to no detection of O_2_^-^ and O_2_^-^-derived oxidant production of ethidium (Figure 7). In mice that received either ethanol or poly I:C treatment, there was a significant increase in the production of O_2_^-^ and O_2_^-^-derived oxidants 27 hours after the last dose of ethanol or three hours after poly I:C administration. Pretreatment with ethanol increased poly I:C production of O_2_^-^ and O_2_^-^-derived oxidants 3 to 4 fold above poly I:C alone and more than 10 fold over controls (Figure 7). These findings indicate that ethanol increases expression of NOX gp91^phox^ and the formation of reactive oxygen species by TLR3 agonist.

### Ethanol increases brain TLR3 and HMGB1

**Figure 8:**
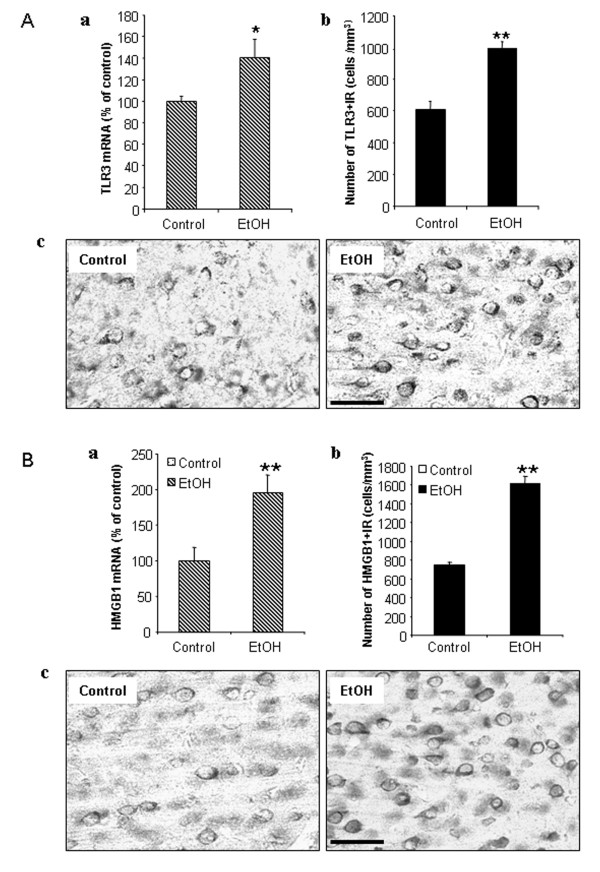
** Ethanol increases TLR3 and HMGB1 expression.** Chronic ethanol treatment of C57BL/6 mice (5 g/kg, i.g., daily for 10 days) increased mRNA and protein expression (+IR) of brain TLR3 and HMGB1. **(A)** Quantitation of TLR3 mRNA and TLR3 + IR. **(A-a)** Level of brain TLR3 mRNA 27 hours following the last dose of ethanol treatment was measured using real-time PCR as described in the methods. Ethanol exposure significantly increased brain TLR3 mRNA. **(A-b)** TLR3 + IR cells were counted in mouse cortex after TLR3 immunostaining. Ethanol significantly increased the number of TLR3 + IR cells. **(A-c)** Representative images of immunohistochemical staining for TLR3 in the cortex of control and ethanol-treated mice. **(B)** Quantitation of HMGB1 mRNA and HMGB1 + IR. **(B-a)** HMGB1 mRNA was measured by real-time PCR in which ethanol increased by about 2 fold. **(B-b)** Quantitative evaluation of HMGB1 + IR. The number of HMGB1 + IR cells was increased about 2 fold. **(B-c)** The representative images of immunohistochemical staining for HMGB1 in the cortex of control and ethanol-treated mice. *P <0.05, **P <0.01, compared with water control group. Scale bar, 50 μm.

To investigate the mechanisms of ethanol proinflammatory responses we measured the expression of TLR3 and HMGB1, a ubiquitous TLR3 co-agonist. Ethanol treatment increased brain TLR3 mRNA (Figure 8A-a). Toll-like receptor positive immunoreactivity (+IR) provides insight into protein levels and ethanol increased TLR3 + IR cells by at least 2 fold in cortex (Figure 8 A-b). Cells with upregulated TLR3 expression appear to be neurons, which are consistent with previous findings by others that TLR3 was expressed in many cell types of the brain in mice [18], including neurons [41], microglia [9] and astrocytes [23]. These measurements were assessed 24 hours after the last ethanol dose indicating that TLR increases persist after blood ethanol concentrations return to zero and during the poly I:C responses. HMGB1 can bind to and activate multiple TLR receptors [42] being required for TLR3 receptor activation [22]. Ethanol treatment increased both brain HMGB1 mRNA (Figure 8B-a) and HMGB1 + IR (Figure 8B-b) by about 2 fold in cortex. Thus, ethanol treatment increased both HMGB1 and TLR3 receptors in brain (Figure 8).

### Increased TLR3 proinflammatory responses increase neurodegeneration

**Figure 9:**
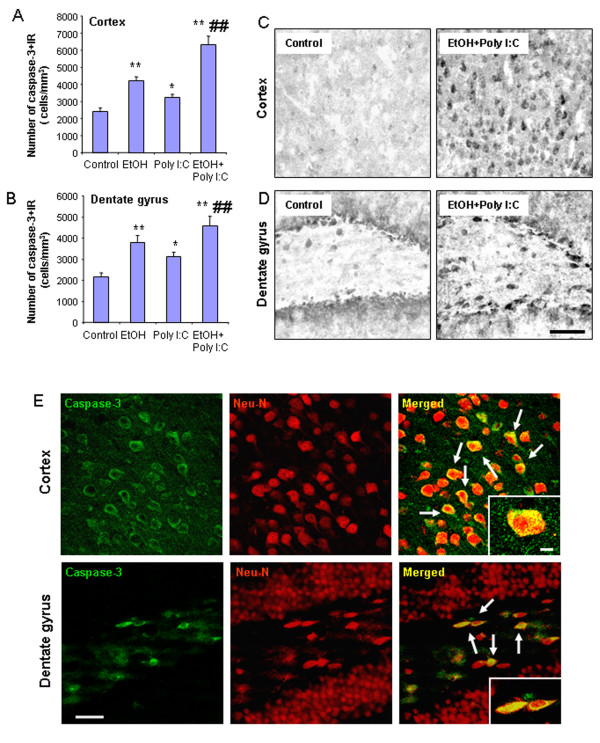
** Activated caspase-3 + IR in brain.** Brain sections were stained with polyclonal cleave caspase-3 (Asp175) antibody, a marker of cell death. **(A)** Quantitation of caspase-3 + IR in cortex. The number of caspase-3 + IR cells in cortex was increased by ethanol, poly I:C and sequential ethanol-poly I:C. **(B)** Quantitation of caspase-3 + IR in hippocampal dentate gyrus. The number of caspase-3 + IR cells in dentate gyrus was increased by ethanol, poly I:C and sequential ethanol-poly I:C. The results are the means ± SEM of two independent experiments performed with seven mice per group. *P <0.05, **P <0.01, compared with vehicle control. ^##^*P* <0.01, compared with poly I:C. **(C and D)** Representative images of caspase-3 + IR in cortex (C) and dentate gyrus (D) in vehicle control and ethanol-poly I:C groups. Scale bar, 200 μm. To determine if caspase-3 + IR was within neurons, brain sections were double-stained with NeuN (a neuronal marker). **(E)** Confocal microscopy images of cortex (upper panels) and dentate gyrus (lower panels) in ethanol-poly I:C group. Immunolabeling was visualized by using Alexa Fluor 488 and 555. Confocal microscopy indicates that caspase-3 + IR cells in green (left panels) are NeuN positive in red (middle panels), as shown in the merged images (right panels) with arrows indicating yellow co-labeling of caspase-3 and NeuN. Insets are higher magnification of the merged images. Scale bar, 30 μm; inset 5 μm.

**Figure 10:**
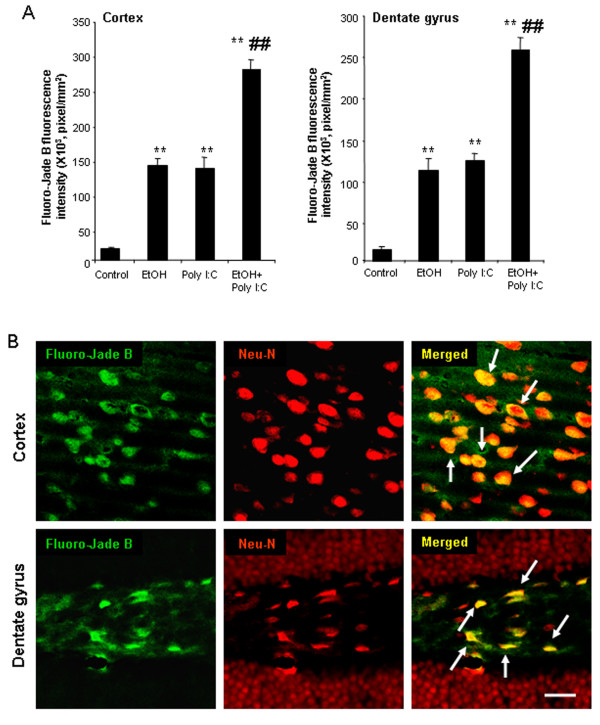
** Activated Fluoro-Jade B in brain. (A)** Brain sections were stained with Fluoro-Jade B, a marker of cell death, and quantitated in cortex and dentate gyrus. The Fluoro-Jade B fluorescence in cortex and dentate gyrus was increased by ethanol, poly I:C and sequential ethanol-poly I:C. The results are the means ± SEM of two independent experiments performed with seven mice per group. **P <0.01, compared with vehicle control. ^##^*P* <0.01, compared with poly I:C. **(B)** Confocal microscopy images of cortex (upper panels) and dentate gyrus (lower panels) in ethanol-poly I:C group. Immunolabeling was visualized by using Alexa Fluor 488 and 555. Confocal microscopy indicates that Fluoro-Jade B in green (left panels) are NeuN positive in red (middle panels), as shown in the merged images (right panels) with arrows indicating yellow co-labeling. Scale bar, 30 μm.

To investigate the relationship among proinflammatory gene induction, microglial activation, oxidative stress, and neuronal cell death, we assessed the cell death markers, activated caspase-3 and Fluoro-Jade B. Ethanol, poly I:C and sequential ethanol-poly I:C increased caspase-3 + IR cells in both cortex and hippocampus (Figure 9). Ethanol-poly I:C caused significantly greater increases in caspase-3 + IR cells than either alone. Double immunohistochemistry with confocal microscopy revealed that caspase-3 + IR was colocalized with NeuN, a neuronal marker, in both cortex and hippocampus (Figure 9E), suggesting neuronal cell death. Fluoro-Jade B, another cell death marker, was also increased by ethanol, poly I:C and sequential ethanol-poly I:C treatment in both cortex and hippocampus (Figure 10). Pretreatment with ethanol more than doubled poly I:C increases in Fluoro-Jade B staining, compared to poly I:C alone group (Figure 10A). Confocal microscopy indicated that most Fluoro-Jade B-positive cells were colocalized with NeuN + IR in both cortex and hippocampal dentate gyrus (Figure 10B). These results indicate that markers of neuronal death are increased by ethanol, poly I:C and sequential ethanol-poly I:C in parallel with induction of neuroinflammatory genes and oxidative stress.

### Inhibition of microglial activation and neurodegeneration

**Figure 11:**
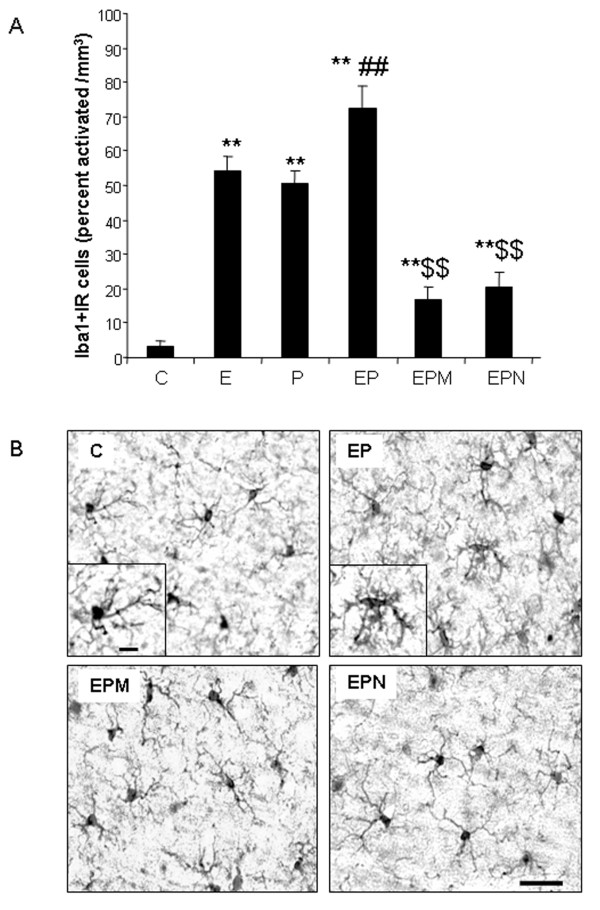
** Minocycline and naltrexone block microglial activation.***(A)* Quantification of activated Iba1 + IR cells in cortex. Ethanol, poly I:C and ethanol-poly I:C treatment groups show increased microglial activation. Minocycline and naltrexone decreased ethanol-poly I:C-activated Iba1 + IR cells. (C, control; E, ethanol; P, poly I:C; EP, ethanol-poly I:C; EPM, ethanol-poly I:C-minocycline; EPN, ethanol-poly I:C-naltrexone.). **(B)** Representative images from vehicle control (C), ethanol-poly I:C (EP), ethanol-poly I:C-minocycline (EPM) and ethanol-poly I:C-naltrexone (EPN) groups in cortex. In control, EPM and EPN groups, most microglia are in a resting state: small cell bodies with thin, highly ramified processes. In the EP-treated group, microglia are activated: large cell bodies, irregular shape and intensified Iba1 staining. **P <0.01, compared with control group. ^##^*P* <0.01, compared with poly I:C group. ^$$^*P* <0.01, compared with ethanol-poly I:C group. Scale bar, 200 μm.

**Figure 12:**
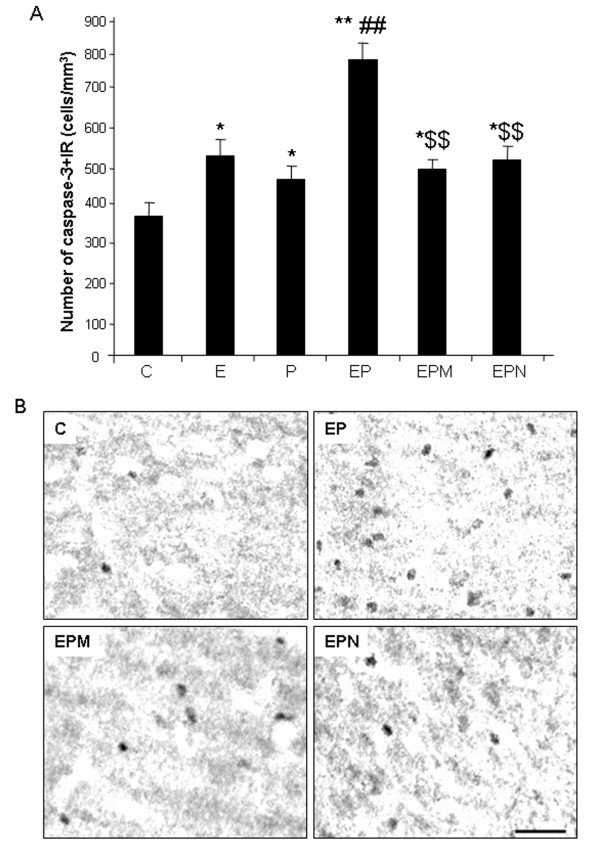
** Minocycline and naltrexone blunt ethanol-poly I:C-induced caspase-3 + IR. (A)** Brain sections were stained with polyclonal cleave caspase-3 (Asp175) antibody. Immunolabeling was visualized by using nickel-enhanced 3,3′-diaminobenzidinne (DAB) as described in the methods. The number of caspase-3 + IR cells in cortex was significantly increased in ethanol, poly I:C and ethanol-poly I:C treatment groups. Minocycline and naltrexone reduced ethanol-poly I:C-induced caspase-3 expression. (C, control; E, ethanol; P, poly I:C; EP, ethanol-poly I:C; EPM, ethanol-poly I:C-minocycline; EPN, ethanol-poly I:C-naltrexone). **(B)** Images are representative of vehicle control (C), ethanol-poly I:C (EP), ethanol-poly I:C-minocycline (EPM) and ethanol-poly I:C-naltrexone (EPN) groups in cortex. Scale bar, 50 μm. *P <0.05, **P <0.01, compared with vehicle control. ^##^*P* <0.01, compared with poly I:C. ^$$^*P* <0.01, compared with ethanol-poly I:C.

Microglia are the innate immune cells of brain and are known to initiate neuroinflammatory responses [6]. Iba1 + IR provides a morphological assessment of microglial activation that parallels the progressive proinflammatory oxidative stress responses in brain to ethanol, poly I:C and ethanol-poly I:C (Figures 4 and 5). Minocycline, an antibiotic known to block microglial activation [43], was used to investigate the link between proinflammatory microglial activation and markers of neuronal death. Minocycline treatment blocked ethanol-poly I:C-induced increases in microglial Iba1 + IR (Figure 11) as well as caspase-3 + IR (Figure 12) suggesting microglial proinflammatory activation contributes to neuronal death. Recent studies have found that the opiate receptor antagonist naltrexone has anti-inflammatory actions that, in part, are related to binding to TLR receptors [44,45]. We found that naltrexone blocked ethanol-poly I:C increased microglial activation and expression of activated caspase-3, a cell death marker (Figures 11 and 12). These findings indicate that minocycline and naltrexone inhibition of microglial activation by ethanol, poly I:C and ethanol pretreatment potentiated proinflammatory responses blunt ethanol-poly I:C potentiated neurodegeneration.

## Discussion

**Figure 13:**
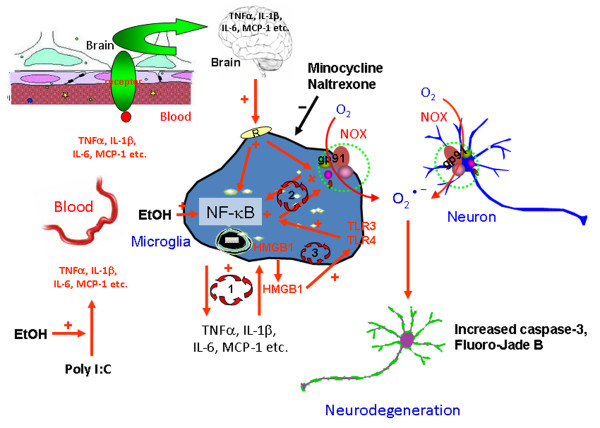
**Schematic summary and hypothetical mechanisms of neuroinflammation and neurodegeneration.** (Lower left) Chronic ethanol treatment potentiates poly I:C increases serum TNFα IL-1β, IL-6 and MCP-1 protein. These proteins in the blood enter the brain through transport systems or other mechanisms as described in the discussion (upper left). In brain these proinflammatory cytokines activate microglia. Ethanol can also directly activate NF-κB transcription. Activated microglia amplify the brain neuroinflammatory response through at least three potential mechanisms. Loop 1 represents microglial synthesis and release of cytokines that activate transcription factor NF-κB to synthesize and release more inflammatory cytokines, which further activates the microglia, producing more proinflammatory signals. Loop 2 involves activation of NADPH oxidase (NOX) in microglia that produces reactive oxygen species that activate transcription factor NF-κB to synthesize and release more inflammatory cytokines. Loop 3 involves HMGB1, a TLR activator, and TLR3 on microglia that stimulates NF-κB and microglial activation. Cytokine, glutamate and/or ethanol release of HMGB1 that can activate multiple TLR receptors on microglia. Our findings of ethanol increased HMGB1 and TLR3 expression in brain support a role for loop 3 in microglial activation. Together, these amplify proinflammatory responses that spread from microglia to neurons (upper right). Neuronal expression of NOX increases oxidative stress leading to neuronal death. Minocycline and naltrexone block microglial activation and blunt neuronal death. These studies suggest that blood proinflammatory signals contribute to neuroinflammation and neurodegeneration that can be prevented by blocking microglial proinflammatory activation

We report here that intraperitoneal poly I:C when sensitized with d-GalN increased cytokines (TNFα, IL-1β, IL-6) and the cytokine-chemokine (MCP-1) in both blood and brain. Either poly I:C or d-GalN alone did not elevate serum and brain TNFα levels (data not shown), which is consistent with previous studies [4,46]. The three-day time course indicated poly I:C-induced brain and blood TNFα peaked at approximately three hours. Previously we found that intraperitoneal LPS, a toll-like receptor 4 (TLR4) agonist, increases liver, brain and blood TNFα that peaked one hour after treatment [1]. Increases in blood TNFα by TNFα injection or induced through LPS treatment required blood–brain barrier TNFα receptor-mediated transport to fully activate brain neuroinflammatory responses [1]. Many tissues may release cytokines into blood, spreading proinflammatory responses to other tissues. The liver and gut have large numbers of monocyte-like cells making it likely they release cytokines into the blood. Although poly I:C-stimulated brain and blood TNFαpeaked at the same time, blood levels returned to near zero by one day, whereas brain TNFα levels remained elevated for three days, the longest time point studied. We found a single LPS injection induced a blood response of less than 24 hours, whereas the brain neuroinflammatory response lasted for more than 10 months [1], consistent with the hypothesis that increases in blood proinflammatory cytokines trigger a persistent increase in neuroinflammation. A delayed increase in liver anti-inflammatory IL-10 may contribute to loss of systemic responses, whereas brain shows a delayed decrease in IL-10, possibly contributing to persistent brain neuroinflammation [27]. In this study, we investigated TNFα, IL-1β, IL-6 and MCP-1 in both blood and brain across four treatment groups that provided graded responses increasing in magnitude, for example, low controls, small ethanol alone responses, significant poly I:C responses and the largest response from ethanol-poly I:C treatment. For example, serum MCP-1 and brain MCP-1 mRNA and protein increase in parallel from controls that are a fewfold less than ethanol alone, with poly I:C alone manyfold larger and sequential ethanol-poly I:C treatment being significantly more than any other treatments. We also found that microglia, the innate immune cells of brain, showed morphological activation that paralleled the level of proinflammatory gene induction across control, ethanol, poly I:C and ethanol-poly I:C groups consistent with microglia responding to blood proinflammatory signals and amplifying the responses. We show here that TNFα, IL-1β, IL-6, and MCP-1 each shows graded increases in blood that resemble graded increases in brain mRNA and protein as well as stages of microglial activation across treatment groups. Proinflammatory cytokines have a blood-to-brain saturable transport system that carries cytokines and chemokines across the blood–brain barrier into brain [41]. Increased mRNA indicates brain protein increases are likely both synthesis and transport. Microglia are the innate immune cells of brain that express cytokine and TLR receptors that respond to many immune signals including cytokines and endogenous TLR agonists, such as HMGB1, a ubiquitous protein and TLR receptor agonist [42,47,48]. Microglia are uniquely sensitive to the brain environment and are thought to initiate neuroinflammatory responses [6]. This is consistent with our finding of increasing morphological activation of microglia coinciding with induction of brain TNFα, IL-1β, IL-6, and MCP-1 mRNA and blood protein levels of these cytokines and chemokines (Figure 13). However, endothelial cells in brain form the blood–brain barrier and both transport and increase synthesis and secretion of cytokines into brain [49]. Our findings are consistent with increases in blood proinflammatory cytokines contributing to activation of brain microglia, endothelial cells and neuroinflammatory gene induction in multiple types of brain cells (See Figure 13).

αβκκκκProinflammatory responses expand through paracrine and autocrine amplification, for example, within the initially activated, such as microglia, as well as across adjacent cells through signals that converge upon NF-κB activating transcription [3,50]. TNFα receptors, IL-1β receptors, poly I:C-TLR3 receptors and LPS-TLR4 receptors activate kinases cascades that increase NF-κB transcription [51]. We found increased brain TNFα, IL-1β, IL-6, MCP-1 and NOX mRNA consistent with activation of NF-κB transcription. Resting microglia express TLR and cytokine receptors that can respond to cytokines entering the brain and contribute to proinflammatory amplification in brain. Increases in brain cytokines transported from blood and/or synthesized within brain likely contribute to brain responses (Figure 13).

At least two other mechanisms may contribute to brain responses, oxidative stress and HMGB1-TLR signaling. NF-κB transcription is also activated by reactive oxygen species and we found increased expression of NOX and superoxide, another possible mechanism of amplification of brain proinflammatory responses. Yet another mechanism could involve HMGB1, a ubiquitous cytokine-like protein that is an agonist or co-agonist across multiple TLR and other innate immune receptors. HMGB1 is released by hyper-excitability, cytokines, cell damage, TLR receptor activation and other stimuli [52] (Figure 13). Recent studies have found that HMGB1 is required for TLR3 receptor signaling [22]. HMGB1 release and activation of TLR receptors represent another mechanism of brain proinflammatory amplification in addition to oxidative stress and brain cytokine increases.

We found that ethanol increased brain expression of HMGB1 and TLR3 as well as potentiating poly I:C induction of proinflammatory genes. *In vitro* studies have found ethanol increased NF-κB transcription in brain slice cultures [3,50,53], astrocytes [54] and microglial cultures [55]. Ethanol activation mimics LPS-TLR4 induction of neuroinflammation [27] and is blunted in mice lacking TLR4 receptors [27,30]. Consistent with ethanol inducing neuroinflammation, studies of postmortem human alcoholic brain have found increased expression of inflammatory genes and genes linked to increased NF-κB-DNA [56], as well as increased histochemical microglial markers and brain levels of the chemokine MCP-1 [25]. These findings indicate ethanol and poly I:C-TLR3 signaling activate NF-κB transcription consistent with ethanol priming microglia with mild activation, perhaps related to induction of HMGB1 and TLR3, that results in increased poly I:C microglial activation and induction of proinflammatory genes.

Many neurodegenerative diseases share increased oxidative stress, increased NOX and increased TLR expression [40]. We have previously found in mice that neuroinflammatory responses that increase NOX expression and levels of reactive oxygen species such as superoxide are linked to neurodegeneration [26]. Alcohol-induced neurodegeneration is found in frontal cortex and hippocampus in rodent models [20] and humans [21]. We found increased morphological microglial activation, NADPH oxidase gp91^phox^ and superoxide levels, as well as activated caspase-3 and Fluoro-Jade B markers of neurodegeneration in both cortex and hippocampal dentate gyrus. Ethanol-poly I:C treatment had significantly higher levels of NOX gp91^phox^ mRNA, NOX gp91^phox^ + IR protein, superoxide levels and cell death markers. NOX expression was co-localized with markers of neurons and microglia. However, caspase-3 + IR cells and Fluoro-Jade B markers of cell death were predominantly found in NeuN + IR neurons. We have found human postmortem alcoholic brain has increased neuronal expression of NOX gp91^phox^ + IR [26]. These studies are consistent with microglial proinflammatory amplification causing neuronal induction of NOX, increasing oxidative stress that causes neuronal death and neurodegeneration.

To further investigate the role of microglial activation in neurodegeneration, we studied minocycline and naltrexone. Although the exact molecular mechanisms are unknown, minocycline is well characterized as an inhibitor of brain microglial activation [43]. Naltrexone is known to alter neuroinflammatory responses [44,45]. Interestingly, neuroinflammation appears to be linked to addiction [3]. Systemic endotoxin treatment leads to a delayed persistent increase in ethanol drinking by mice [57], whereas transgenic mice lacking proinflammatory genes show reduced ethanol drinking [58] and altered acute ethanol motor and sedative effects [59]. In addition, viral vector siRNA knock-down of TLR4 in amygdala reduces lever pressing for alcohol in alcohol-dependent rats [60]. Naloxone, which is similar to naltrexone, has been reported to block NADPH oxidase [61]. Naltrexone has been recently found to block TLR responses [45]. These studies are consistent with naltrexone having anti-inflammatory effects. Naltrexone is known to reduce drinking in both animals and humans, and is used to treat human alcoholism [62]. Interestingly, minocycline also reduces ethanol drinking in rats [63]. In the present study, we found both minocycline and naltrexone reduced ethanol-poly I:C-elicited microglial activation and increased caspase-3 + IR cells. These findings support the hypothesis that proinflammatory microglial activation contributes to neurodegeneration.

In summary, the findings presented support a connection between blood and brain proinflammatory responses, with the magnitude of peak blood proinflammatory cytokines being reflected in the degree of brain microglial activation and neuroinflammatory responses. Multiple mechanisms converge upon microglial activation that contributes to neurodegeneration (Figure 13). Proinflammatory amplification induced neuronal NADPH oxidase, superoxide formation and increased markers of neuronal death. Naltrexone and minocycline block microglial activation and neurodegeneration supporting the role of microglia contributing to neurodegeneration.

## Abbreviations

CCL-2, chemokine (C-C motif) ligand 2; CNS, central nervous system; ELISA, enzyme-linked immunosorbent assay; HMGB1, high mobility group box 1; IACUC, Institutional Animal Care and Use Committee; i.g., intragastrically; IL-1β, interleukin-1; i.p, intraperitoneally; IR, immunoreactivity; L-DOPA, L-3,4-dihydroxyphenylalanine; LPS, lipopolysaccharide; MCP-1, monocyte chemotactic protein-1; NF-κB, nuclear factor-kappa B; PBS, phosphate buffered saline; PCR, polymerase chain reaction; poly I:C, polyinosine-polycytidylic acid; ROS, reactive oxygen species; siRNA, small interfering RNA; TLR, toll-like receptor; TNFα, tumor necrosis factor-α.

## Competing interests

The authors declare that they have no competing interests.

## Authors’ contributions

LQ participated in the experimental design, performed animal experiments and data analysis, and drafted the manuscript. FTC conceived the study, assisted with its design and data analysis, and helped draft the manuscript. All authors read and approved the final manuscript.

## References

[B1] Qin L Wu X Block ML Liu Y Breese GR Hong JS Knapp DJ Crews FT Systemic LPS causes chronic neuroinflammation and progressive neurodegeneration Glia 2007 55 453 462 10.1002/glia.20467 17203472 PMC2871685

[B2] Liu Y Qin L Wilson B Wu X Qian L Granholm AC Crews FT Hong JS Endotoxin induces a delayed loss of TH-IR neurons in substantia nigra and motor behavioral deficits Neurotoxicology 2008 29 864 870 10.1016/j.neuro.2008.02.014 18471886 PMC2762082

[B3] Crews FT Zou J Qin L Induction of innate immune genes in brain create the neurobiology of addiction Brain Behav Immun 2011 Suppl 1 S4 S12 21402143 10.1016/j.bbi.2011.03.003 PMC3552373

[B4] Jiang W Sun R Wei H Tian Z Toll-like receptor 3 ligand attenuates LPS-induced liver injury by down-regulation of toll-like receptor 4 expression on macrophages Proc Natl Acad Sci USA 2005 102 17077 17082 10.1073/pnas.0504570102 16287979 PMC1287976

[B5] Finch CE Morgan TE Systemic inflammation, infection, ApoE alleles, and Alzheimer disease: a position paper Curr Alzheimer Res 2007 4 185 189 10.2174/156720507780362254 17430245

[B6] Block ML Zecca L Hong JS Microglia-mediated neurotoxicity: uncovering the molecular mechanisms Nat Rev Neurosci 2007 8 57 69 10.1038/nrn2038 17180163

[B7] Doyle SE O’Connell R Vaidya SA Chow EK Yee K Cheng G Toll-like receptor 3 mediates a more potent antiviral response than Toll-like receptor 4 J Immunol 2003 170 3565 3571 12646618 10.4049/jimmunol.170.7.3565

[B8] Alexopoulou L Holt AC Medzhitov R Flavell RA Recognition of double-stranded RNA and activation of NF-kappaB by Toll-like receptor 3 Nature 2001 413 732 738 10.1038/35099560 11607032

[B9] Olson JK Miller SD Microglia initiate central nervous system innate and adaptive immune responses through multiple TLRs J Immunol 2004 173 3916 3924 15356140 10.4049/jimmunol.173.6.3916

[B10] Re F Strominger JL IL-10 released by concomitant TLR2 stimulation blocks the induction of a subset of Th1 cytokines that are specifically induced by TLR4 or TLR3 in human dendritic cells J Immunol 2004 173 7548 7555 15585882 10.4049/jimmunol.173.12.7548

[B11] Sivori S Falco M Della Chiesa M Carlomagno S Vitale M Moretta L Moretta A CpG and double-stranded RNA trigger human NK cells by Toll-like receptors: induction of cytokine release and cytotoxicity against tumors and dendritic cells Proc Natl Acad Sci U S A 2004 101 10116 10121 10.1073/pnas.0403744101 15218108 PMC454174

[B12] Scumpia PO Kelly KM Reeves WH Stevens BR Double-stranded RNA signals antiviral and inflammatory programs and dysfunctional glutamate transport in TLR3-expressing astrocytes Glia 2005 52 153 162 10.1002/glia.20234 15920723

[B13] Chen CJ Chen JH Chen SY Liao SL Raung SL Upregulation of RANTES gene expression in neuroglia by Japanese encephalitis virus infection J Virol 2004 78 12107 12119 10.1128/JVI.78.22.12107-12119.2004 15507597 PMC525064

[B14] Kielian T Microglia and chemokines in infectious diseases of the nervous system: views and reviews Front Biosci 2004 9 732 750 10.2741/1266 14766404

[B15] Guha-Thakurta N Majde JA Early induction of proinflammatory cytokine and type I interferon mRNAs following Newcastle disease virus, poly [rI:rC], or low-dose LPS challenge of the mouse J Interferon Cytokine Res 1997 17 197 204 10.1089/jir.1997.17.197 9142648

[B16] Schulz O Diebold SS Chen M Naslund TI Nolte MA Alexopoulou L Azuma YT Flavell RA Liljestrom P Reis e Sousa C Toll-like receptor 3 promotes cross-priming to virus-infected cells Nature 2005 433 887 892 10.1038/nature03326 15711573

[B17] Park C Lee S Cho IH Lee HK Kim D Choi SY Oh SB Park K Kim JS Lee SJ TLR3-mediated signal induces proinflammatory cytokine and chemokine gene expression in astrocytes: differential signaling mechanisms of TLR3-induced IP-10 and IL-8 gene expression Glia 2006 53 248 256 10.1002/glia.20278 16265667

[B18] Carty M Bowie AG Evaluating the role of Toll-like receptors in diseases of the central nervous system Biochem Pharmacol 2011 81 825 837 10.1016/j.bcp.2011.01.003 21241665

[B19] Bsibsi M Ravid R Gveric D van Noort JM Broad expression of Toll-like receptors in the human central nervous system J Neuropathol Exp Neurol 2002 61 1013 1021 12430718 10.1093/jnen/61.11.1013

[B20] Crews FT Nixon K Mechanisms of neurodegeneration and regeneration in alcoholism Alcohol Alcohol 2009 44 115 127 18940959 10.1093/alcalc/agn079 PMC2948812

[B21] Harper C The neuropathology of alcohol-related brain damage Alcohol Alcohol 2009 44 136 140 19147798 10.1093/alcalc/agn102

[B22] Yanai H Ban T Wang Z Choi MK Kawamura T Negishi H Nakasato M Lu Y Hangai S Koshiba R Savitsky D Ronfani L Akira S Bianchi ME Honda K Tamura T Kodama T Taniguchi T HMGB proteins function as universal sentinels for nucleic-acid-mediated innate immune responses Nature 2009 462 99 103 10.1038/nature08512 19890330

[B23] Carpentier PA Begolka WS Olson JK Elhofy A Karpus WJ Miller SD Differential activation of astrocytes by innate and adaptive immune stimuli Glia 2005 49 360 374 10.1002/glia.20117 15538753

[B24] Nelson S Kolls JK Alcohol, host defence and society Nat Rev Immunol 2002 2 205 209 10.1038/nri744 11913071

[B25] He J Crews FT Increased MCP-1 and microglia in various regions of the human alcoholic brain Exp Neurol 2008 210 349 358 10.1016/j.expneurol.2007.11.017 18190912 PMC2346541

[B26] Qin L Crews FT NADPH oxidase and reactive oxygen species contribute to alcohol-induced microglial activation and neurodegeneration J Neuroinflammation 2012 9 5 10.1186/1742-2094-9-5 22240163 PMC3271961

[B27] Qin L He J Hanes RN Pluzarev O Hong JS Crews FT Increased systemic and brain cytokine production and neuroinflammation by endotoxin following ethanol treatment J Neuroinflammation 2008 5 10 10.1186/1742-2094-5-10 18348728 PMC2373291

[B28] Pruett SB Fan R Zheng Q Acute ethanol administration profoundly alters poly I:C-induced cytokine expression in mice by a mechanism that is not dependent on corticosterone Life Sci 2003 72 1825 1839 10.1016/S0024-3205(02)02507-9 12586220

[B29] Mandrekar P Catalano D Szabo G Inhibition of lipopolysaccharide-mediated NFkappaB activation by ethanol in human monocytes Int Immunol 1999 11 1781 1790 10.1093/intimm/11.11.1781 10545482

[B30] Alfonso-Loeches S Pascual-Lucas M Blanco AM Sanchez-Vera I Guerri C Pivotal role of TLR4 receptors in alcohol-induced neuroinflammation and brain damage J Neurosci 2010 30 8285 8295 10.1523/JNEUROSCI.0976-10.2010 20554880 PMC6634595

[B31] Qin L Liu Y Wang T Wei SJ Block ML Wilson B Liu B Hong JS NADPH oxidase mediates lipopolysaccharide-induced neurotoxicity and proinflammatory gene expression in activated microglia J Biol Chem 2004 279 1415 1421 14578353 10.1074/jbc.M307657200

[B32] Bindokas VP Jordan J Lee CC Miller RJ Superoxide production in rat hippocampal neurons: selective imaging with hydroethidine J Neurosci 1996 16 1324 1336 8778284 10.1523/JNEUROSCI.16-04-01324.1996 PMC6578569

[B33] Wu DC Teismann P Tieu K Vila M Jackson-Lewis V Ischiropoulos H Przedborski S NADPH oxidase mediates oxidative stress in the 1-methyl-4-phenyl-1,2,3,6-tetrahydropyridine model of Parkinson’s disease Proc Natl Acad Sci USA 2003 100 6145 6150 10.1073/pnas.0937239100 12721370 PMC156340

[B34] Crews FT Nixon K Wilkie ME Exercise reverses ethanol inhibition of neural stem cell proliferation Alcohol 2004 33 63 71 15353174 10.1016/j.alcohol.2004.04.005

[B35] McClain JA Morris SA Deeny MA Marshall SA Hayes DM Kiser ZM Nixon K Adolescent binge alcohol exposure induces long-lasting partial activation of microglia Brain Behav Immun 2011 Suppl 1 S120 S128 21262339 10.1016/j.bbi.2011.01.006 PMC3098298

[B36] Chen WJ Parnell SE West JR Neonatal alcohol and nicotine exposure limits brain growth and depletes cerebellar Purkinje cells Alcohol 1998 15 33 41 10.1016/S0741-8329(97)00084-0 9426835

[B37] Gundersen HJ Bendtsen TF Korbo L Marcussen N Moller A Nielsen K Nyengaard JR Pakkenberg B Sorensen FB Vesterby A West MJ Some new, simple and efficient stereological methods and their use in pathological research and diagnosis APMIS 1988 96 379 394 10.1111/j.1699-0463.1988.tb05320.x 3288247

[B38] West MJ Gundersen HJ Unbiased stereological estimation of the number of neurons in the human hippocampus J Comp Neurol 1990 296 1 22 10.1002/cne.902960102 2358525

[B39] Gu L Okada Y Clinton SK Gerard C Sukhova GK Libby P Rollins BJ Absence of monocyte chemoattractant protein-1 reduces atherosclerosis in low density lipoprotein receptor-deficient mice Mol Cell 1998 2 275 281 10.1016/S1097-2765(00)80139-2 9734366

[B40] Glass CK Saijo K Winner B Marchetto MC Gage FH Mechanisms underlying inflammation in neurodegeneration Cell 2010 140 918 934 10.1016/j.cell.2010.02.016 20303880 PMC2873093

[B41] Tang SC Arumugam TV Xu X Cheng A Mughal MR Jo DG Lathia JD Siler DA Chigurupati S Ouyang X Magnus T Camandola S Mattson MP Pivotal role for neuronal Toll-like receptors in ischemic brain injury and functional deficits Proc Natl Acad Sci USA 2007 104 13798 13803 10.1073/pnas.0702553104 17693552 PMC1959462

[B42] Garg AD Nowis D Golab J Vandenabeele P Krysko DV Agostinis P Immunogenic cell death, DAMPs and anticancer therapeutics: an emerging amalgamation Biochim Biophys Acta 2010 1805 53 71 19720113 10.1016/j.bbcan.2009.08.003

[B43] Plane JM Shen Y Pleasure DE Deng W Prospects for minocycline neuroprotection Arch Neurol 2010 67 1442 1448 10.1001/archneurol.2010.191 20697034 PMC3127230

[B44] Hutchinson MR Shavit Y Grace PM Rice KC Maier SF Watkins LR Exploring the neuroimmunopharmacology of opioids: an integrative review of mechanisms of central immune signaling and their implications for opioid analgesia Pharmacol Rev 2011 63 772 810 10.1124/pr.110.004135 21752874 PMC3141878

[B45] Hutchinson MR Zhang Y Brown K Coats BD Shridhar M Sholar PW Patel SJ Crysdale NY Harrison JA Maier SF Rice KC Watkins LR Non-stereoselective reversal of neuropathic pain by naloxone and naltrexone: involvement of toll-like receptor 4 (TLR4) Eur J Neurosci 2008 28 20 29 10.1111/j.1460-9568.2008.06321.x 18662331 PMC2588470

[B46] Dejager L Libert C Tumor necrosis factor alpha mediates the lethal hepatotoxic effects of poly(I:C) in D-galactosamine-sensitized mice Cytokine 2008 42 55 61 10.1016/j.cyto.2008.01.014 18331798

[B47] Sims GP Rowe DC Rietdijk ST Herbst R Coyle AJ HMGB1 and RAGE in inflammation and cancer Annu Rev Immunol 2010 28 367 388 10.1146/annurev.immunol.021908.132603 20192808

[B48] Huang W Tang Y Li L HMGB1, a potent proinflammatory cytokine in sepsis Cytokine 2010 51 119 126 10.1016/j.cyto.2010.02.021 20347329

[B49] Banks WA Erickson MA The blood–brain barrier and immune function and dysfunction Neurobiol Dis 2010 37 26 32 10.1016/j.nbd.2009.07.031 19664708

[B50] Zou J Crews F Induction of innate immune gene expression cascades in brain slice cultures by ethanol: key role of NF-kappaB and proinflammatory cytokines Alcohol Clin Exp Res 2010 34 777 789 10.1111/j.1530-0277.2010.01150.x 20201932

[B51] Nguyen MD Julien JP Rivest S Innate immunity: the missing link in neuroprotection and neurodegeneration? Nat Rev Neurosci 2002 3 216 227 10.1038/nrn752 11994753

[B52] Maroso M Balosso S Ravizza T Liu J Aronica E Iyer AM Rossetti C Molteni M Casalgrandi M Manfredi AA Bianchi ME Vezzani A Toll-like receptor 4 and high-mobility group box-1 are involved in ictogenesis and can be targeted to reduce seizures Nat Med 2010 16 413 419 10.1038/nm.2127 20348922

[B53] Zou J Crews F CREB and NF-kappaB transcription factors regulate sensitivity to excitotoxic and oxidative stress induced neuronal cell death Cell Mol Neurobiol 2006 26 385 405 16633891 10.1007/s10571-006-9045-9 PMC11520752

[B54] Blanco AM Valles SL Pascual M Guerri C Involvement of TLR4/type I IL-1 receptor signaling in the induction of inflammatory mediators and cell death induced by ethanol in cultured astrocytes J Immunol 2005 175 6893 6899 16272348 10.4049/jimmunol.175.10.6893

[B55] Fernandez-Lizarbe S Pascual M Guerri C Critical role of TLR4 response in the activation of microglia induced by ethanol J Immunol 2009 183 4733 4744 10.4049/jimmunol.0803590 19752239

[B56] Liu J Lewohl JM Harris RA Iyer VR Dodd PR Randall PK Mayfield RD Patterns of gene expression in the frontal cortex discriminate alcoholic from nonalcoholic individuals Neuropsychopharmacology 2006 31 1574 1582 10.1038/sj.npp.1300947 16292326

[B57] Blednov YA Benavidez JM Geil C Perra S Morikawa H Harris RA Activation of inflammatory signaling by lipopolysaccharide produces a prolonged increase of voluntary alcohol intake in mice Brain Behav Immun 2011 Suppl 1 S92 S105 21266194 10.1016/j.bbi.2011.01.008 PMC3098320

[B58] Blednov YA Ponomarev I Geil C Bergeson S Koob GF Harris RA Neuroimmune regulation of alcohol consumption: behavioral validation of genes obtained from genomic studies Addict Biol 2012 1 108 120 21309947 10.1111/j.1369-1600.2010.00284.x PMC3117922

[B59] Wu Y Lousberg EL Moldenhauer LM Hayball JD Robertson SA Coller JK Watkins LR Somogyi AA Hutchinson MR Attenuation of microglial and IL-1 signaling protects mice from acute alcohol-induced sedation and/or motor impairment Brain Behav Immun 2011 Suppl 1 S155 S164 21276848 10.1016/j.bbi.2011.01.012

[B60] Liu J Yang AR Kelly T Puche A Esoga C June HL Elnabawi A Merchenthaler I Sieghart W Sr June HL Aurelian L Binge alcohol drinking is associated with GABAA alpha2-regulated Toll-like receptor 4 (TLR4) expression in the central amygdala Proc Natl Acad Sci U SA 2011 108 4465 4470 10.1073/pnas.1019020108 PMC3060224 21368176

[B61] Wang Q Zhou H Gao H Chen SH Chu CH Wilson B Hong JS Naloxone inhibits immune cell function by suppressing superoxide production through a direct interaction with gp91phox subunit of NADPH oxidase J Neuroinflammation 2012 9 32 10.1186/1742-2094-9-32 22340895 PMC3305409

[B62] Garbutt JC Efficacy and tolerability of naltrexone in the management of alcohol dependence Curr Pharm Des 2010 16 2091 2097 10.2174/138161210791516459 20482515

[B63] Agrawal RG Hewetson A George CM Syapin PJ Bergeson SE Minocycline reduces ethanol drinking Brain Behav Immun 2011 Suppl 1 S165 S169 21397005 10.1016/j.bbi.2011.03.002 PMC3098317

